# To what extent is Fetal Alcohol Spectrum Disorder considered in policy-related documents in South Africa? A document review

**DOI:** 10.1186/s12961-019-0447-9

**Published:** 2019-04-29

**Authors:** Babatope O. Adebiyi, Ferdinand C. Mukumbang, Anna-Marie Beytell

**Affiliations:** 1 School of Public Health, University of the Western Cape, Robert Sobukwe Road, Bellville, 7535 South Africa; 2 Department of Social Work, University of the Western Cape, Robert Sobukwe Road, Bellville, South Africa

**Keywords:** Fetal alcohol spectrum disorder, policy, guidelines, clauses, South Africa, prevention, management, development disabilities, pregnancy, alcohol

## Abstract

**Background:**

South Africa is considered to have the highest prevalence of fetal alcohol spectrum disorder (FASD) globally. Nevertheless, the extent to which the South African government has responded to the high FASD prevalence at the policy level is unclear. Herein, we aimed to identify targeted and generic clauses that could be attributed to the prevention and management of FASD in relevant South African policy documents.

**Methods:**

We conducted a search of two search engines (PubMed and Google) and the websites of South African national and provincial departments from January to April 2018. A total of 33 policy documents were included in this review. Using content analysis, we sought documents that mention the terms ‘fetal alcohol syndrome’ and ‘fetal alcohol spectrum disorder’. The Framework method was also used to thematically identify specific and generic clauses attributed to the prevention and management of FASD in South Africa.

**Results:**

The content analysis indicated that 12 policy documents contained the searched terms. Findings from the thematic analysis showed that targeted and generic clauses for FASD exist in various policy documents. Some of the generic clauses focused on the regulation of liquor outlets, enforcement of liquor laws, and the general management of persons with mental and educational challenges. Specific clauses focused on creating platforms to improve the awareness, screening, identification and support for individuals with FASD.

**Conclusions:**

There is a noticeable increase in the number of policy documents that considered elements of FASD enacted in the last decade. Although this study revealed the existence of targeted and generic clauses that could be attributed to the prevention and management of FASD, the sustained high prevalence of FASD in South Africa, as reported in the literature, calls for more holistic and comprehensive approaches to tackle the FASD problem in South Africa.

**Electronic supplementary material:**

The online version of this article (10.1186/s12961-019-0447-9) contains supplementary material, which is available to authorized users.

## Background

Fetal alcohol spectrum disorder (FASD) is a group of physical, behavioural and learning conditions that can occur in persons who were exposed to alcohol during pregnancy [1]. According to reports, no amount of alcohol is safe, and there is no safe time to drink during pregnancy that will not lead FASD [2, 3]. Therefore, any amount of alcohol consumed during pregnancy places the foetus at risk.

Globally, 1 in every 13 prenatal alcohol-exposed pregnancies results in FASD, with a global prevalence of 8 per 1000 children and youth in the general population as reported in 2017 [4]. In South Africa, the national prevalence of FASD ranges from 29 to 290 per 1000 live births [5], representing the highest rate globally. In the Western Cape Province, a recently published study estimated the prevalence to be between 196 and 276 children per 1000 [6], representing the highest prevalence in South African provinces.

The high prevalence of FASD recorded in South Africa has been attributed, in part, to the historical drinking culture driven by a system known as the ‘dop system’, whereby farmworkers’ wages were paid in alcoholic beverages [7, 8]. The high prevalence of FASD could also be ascribed to the lack of a comprehensive and multi-sectoral policy. To this end, incessant calls for a coordinated effort for the prevention and management of FASD have been made [5]. The coordinated effort for the prevention and management of FASD requires a sustainable commitment from the government, which could be facilitated by a multi-sectoral approach to policy development.

South Africa has the highest alcohol consumption rate (11 l per capita) in Africa, and is among the highest in the world [9]. The levels of lifetime consumption of alcohol for men and women are 49% and 22%, respectively [10]. Drinking during pregnancy is common in many countries, including South Africa [11], where women consider drinking as a coping strategy for their socioeconomic and sociopolitical realities [12]. In a study conducted in the Cape Metropole of the Western Cape Province, 36.9% of women confirmed that they had consumed alcohol during their current pregnancy or in the 3 months before they knew they were pregnant [13]. In another study conducted in the East Metropole, 22% of the pregnant women in the sample reported using alcohol during pregnancy [14]. This high rate of alcohol consumption is facilitated by the vast presence of illegal liquor stores. For example, in a single province (Western Cape), there are approximately 25,000 illegal liquor stores, known as *shebeens* (home-based taverns), making alcohol readily accessible to people of all ages [15]. The South African government must make a conscious effort to address and tackle this endemic risk factor for FASD [16].

In South Africa, policy-making is in most part the responsibility of the national government, with implementation and monitoring usually occurring at all levels of the government, though mostly at provincial and municipal levels. However, in a few instances, provincial and municipal governments can develop policies to address localised issues. Developing a policy document is an indication of the government’s acknowledgment of a dilemma and acts as a response in the form of an action plan or a strategy to address a specific problem. The policy document usually contains stipulations on the allocation of resources, the timeframe for action and the framework for implementation [17–21]. The development of a National Strategy for HIV, tuberculosis and sexually transmitted infections is a good example of the importance of a policy document in South Africa [22–25]. Despite its shortcomings on implementation, progress has been made towards reaching the 90–90–90 target, especially in achieving a 90% rate of diagnosis in HIV-positive individuals [24]. Learning from the success recorded on HIV, our consideration is that the South African government can achieve some success in curbing the escalating prevalence of FASD in South Africa by developing a comprehensive multi-sectoral policy.

Countries such as Australia, Canada and the United States of America [5, 26–29] have not only recognised FASD as a public health issue but have developed specific FASD guidelines/policies to address it. These guidelines/policies include action plans [30], screening tools [31], frameworks for action [32], diagnosis guidelines [33–35] and treatment improvement protocols [36]. However, in South Africa, the approach to addressing FASD at all levels of government remains non-specific as demonstrated by the presence of generic policies.

In 2008, Rendall-Nkosi et al. [17] conducted a situational analysis to examine the extent to which FASD has been addressed in various South African policy documents. According to these authors, only two policy documents used the term fetal alcohol syndrome (FAS), namely the National Drug Master Plan 2006–2011, and the National Human Genetics Policy Guidelines for the Management & Prevention of Genetic Disorders, Birth Defects & Disabilities. However, other policy documents, including the Guidelines for Maternity Care in South Africa 2002, Education White Paper 5 on Early Childhood Development 2001, and Education White Paper 6 on Inclusive Education 2001, contained blanket or generic clauses that could be attributed to the prevention and management of FASD. The authors also reported that services for individuals with FASD are fragmented among the essential departments (Education, Health, Labour, and Social Development), which may be because of the absence of a multi-sectoral guideline/policy for FASD [17].

Rendall-Nkosi et al. [17], in their situation analysis in 2008, recommended that a further detailed document review in South Africa should be conducted to examine the extent to which FASD is considered in policy documents. To the best of our knowledge, theirs is the only document review on FASD policy reported in the literature. To this end, we conducted another review to examine the extent to which FASD is considered in policy-related documents. We aimed to examine the targeted and blanket clauses that could be attributed to the prevention and management of FASD. These clauses could form part of a proposed guideline policy for the prevention and management of FASD, which is the aim of the larger study [20].

## Methods

### Study design

We conducted a document review with a qualitative approach to analysis [37]. This approach allows for an in-depth examination and interpretation of data to elicit meaning and gain an understanding of a particular issue [38]. Using a document review approach was informed by our aim to identify clauses that speak to the prevention and management of FASD in various relevant South African policies either specifically or in an inclusive manner.

### Identifying relevant documents

We searched PubMed and Google search engines and the websites of South African national and provincial departments, specifically the departments of Education, Health, Social Development, and Trade and Industry, from January to April 2018. We applied the following standard Boolean phrase during the searches: [‘foetal alcohol spectrum disorder’ OR ‘alcohol-related neurodevelopmental disorder’ OR ‘foetal alcohol syndrome’ AND ‘policy’ OR ‘guideline’ OR ‘gazette’ OR ‘action plan’ OR ‘white paper’ OR ‘green paper’ AND ‘South Africa’]. Of note, the use of foetal during the search also captured documents using ‘fetal’. In addition to the online searches, we contacted the departmental designated contact persons in the Departments of Education, Health, Social Development, and Trade and Industry by email to request other policy documents, which may not be available online. These departments were selected because they have policy documents with clauses that could be attributed to the prevention and management of FASD or regulations of alcohol. We also searched references of the selected relevant policy documents for additional related information.

Herein, we considered a policy document as an action plan or a guideline that contains the intention of a government (national or provincial) concerning a particular issue. The search terms were identified and defined by two of the authors (BOA and FCM). BOA conducted the electronic search following the defined search terms and contacted other stakeholders for relevant documents. BOA and FCM finalised the list of documents that met the inclusion criteria. AMB supervised the work.

#### Inclusion criteria

- The document must be about South African policy.
- The document must contain clauses specifically targeting FASD or be attributed to FASD as well as clauses that address alcohol drinking in general.
- If the documents were published in series, the most recent series was considered.

Our initial search yielded 371 articles and documents. Following deduplication, we obtained 152 articles and documents. After screening the titles, executive summaries/abstract and the full texts, we obtained 33 relevant policy documents included for analysis. The list of the documents and their descriptions are presented in Additional file 1.

### Data analysis

The data were analysed using both content analysis [39] and the Framework method [40], a family of thematic analysis [41]. During content analysis, we specifically searched for the terms FAS and FASD. To do this, the first author (BOA) typed each of the terms into the search console of the PDF file of each policy document to identify the occurrence of FAS, FASD or both.

**Fig. 1:**
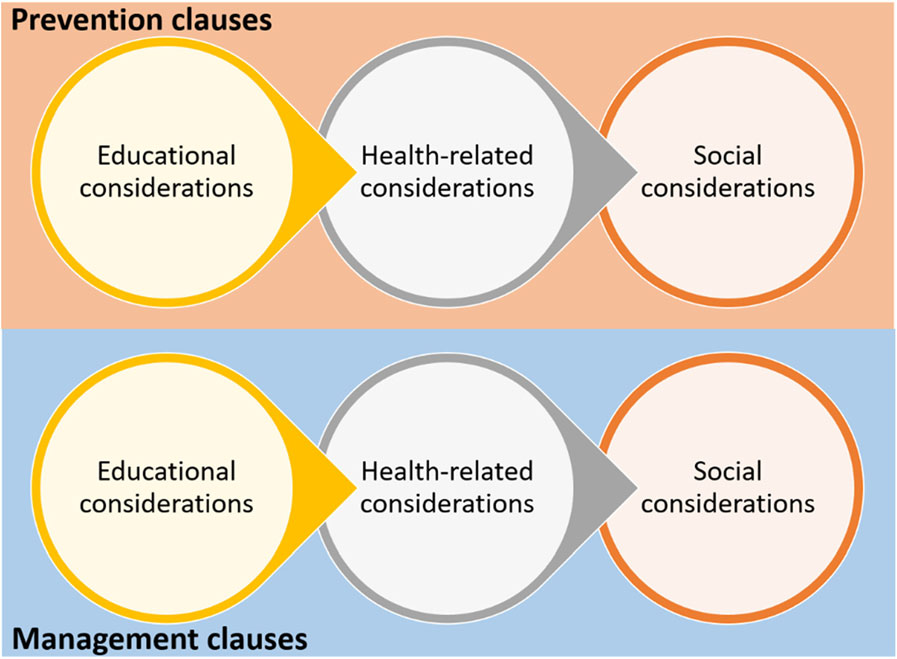
A heuristic framework guiding the document analysis

The thematic analysis entailed reading and re-reading the identified policy documents for familiarisation. Coding of relevant clauses was performed inductively to generate initial codes, which were re-organised to obtain refined codes. In the thematic analysis, we read all the selected documents to identify clauses targeted or attributed to FASD. These clauses, usually sentence(s), were then chatted into an analytical framework (Fig. 1). The framework revealed a classification of the FASD clauses into the main categories of prevention and management. Each category was further subdivided into education, health and social considerations. In a discursive process, consensus was reached between the authors on the fitting of the various clauses under the appropriate themes and subthemes within the analytical framework.

### Data management

For easy identification, we coded the policy documents as DRX, wherein DR stands for policies, action plans or guidelines, and X denotes an arbitrary number from 1 to 33. Targeted clauses are clauses that directly address FASD-related issues in the selected policy documents. Blanket clauses are clauses that could be ascribed to the prevention and management of FASD in the selected policy documents but could be relevant to other conditions. Also described as a generic clause, a blanket clause is considered to be attributed to FASD if it is meant for other conditions, but the clause can also be related to addressing any of the FASD outcomes. While reporting on the specific clauses and blanket clauses attributed to FASD (Fig. 1), we selected only some of the clauses to describe in the Results section. The complete set of clauses is presented as Additional file 2.

## Results

**Fig. 2:**
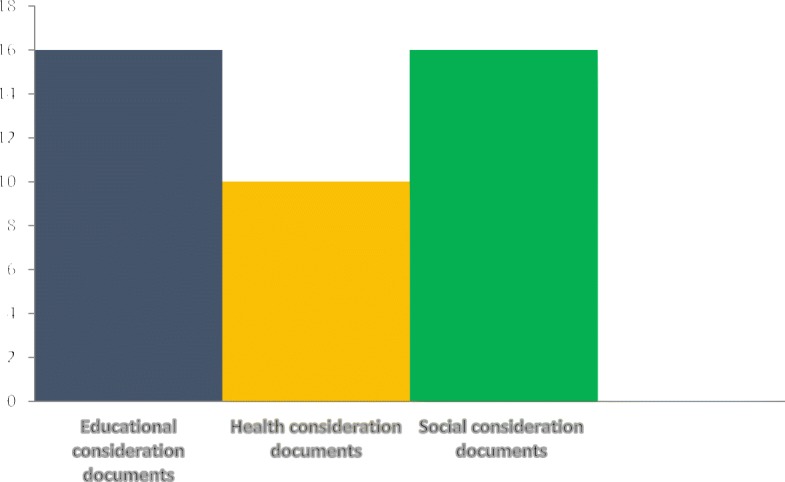
Number of documents that contained each aspect of FASD prevention and management

**Fig. 3:**
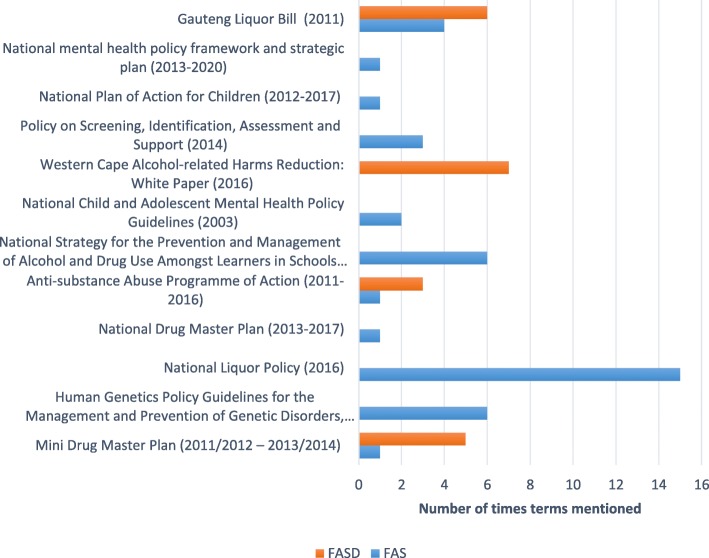
List of documents that contained the terms FAS and FASD

Figure 2 denotes the documents containing targeted or blanket clauses for FASD. Content analysis indicated that 12 policy documents contained the terms FAS and FASD, as illustrated in Fig. 3.

### Presentation of targeted and blanket clauses of FASD

We present the targeted and blanket clauses following our heuristic framework (Fig. 1).

### Targeted clauses

#### Prevention-targeted clauses

Two policy documents contained educational considerations specifically targeting the prevention of FASD. The documents include (1) the Human Genetics Policy Guidelines for the Management and Prevention of Genetic Disorders, Birth Defects and Disabilities [70], and (2) the Western Cape Alcohol-related Harms Reduction: White Paper [71]. Focus on education in these documents is captured in the following two clauses.

“*The continuation of education programmes on FASD. The Western Cape Government (WCG) will continue to focus on education programmes on FASD in collaboration with strategic partners specialising in the field, with the aim of expanding the programmes. Current initiatives include screening participants and providing psychosocial therapy and life-skills training*.” (DR18: page 62)

“*Educate all women regarding the deleterious effects of alcohol on the foetus. Educate all women to avoid alcohol throughout pregnancy*.” (DR2: page 18)

Three policy documents contained health-related clauses specifically addressing the prevention of FASD. These include (1) the Human Genetics Policy Guidelines for the Management and Prevention of Genetic Disorders, Birth Defects, and Disabilities [70]; (2) the Western Cape Alcohol-related Harms Reduction: White Paper [71], and (3) the National Child and Adolescent Mental Health Policy Guidelines [72]. Below, we share clauses selected from some of these documents.

“*Public efforts to improve health, nutrition, education and self-reliance, particularly of women; avoidance of unintended pregnancies, and proper birth spacing through access to contraception and other methods of family planning; improved access to, and quality of, prenatal care and genetic counselling; avoidance of exposure to teratogens (e.g. alcohol) during pregnancy*.” (DR2: page 15)

“*Improve the detection rate for alcohol and other drug abuse at antenatal clinics, and provide the appropriate services to reduce the incidence of fetal alcohol syndrome*.” (DR11: page 21)

Clauses of social consideration mainly targeting the prevention of FASD were found in (1) the Mini Drug Master Plan [73], and (2) the Human Genetics Policy Guidelines for the Management and Prevention of Genetic Disorders, Birth Defects and Disabilities [70].

“*Increase capacity for prevention, identification and development of appropriate interventions for individuals and families affected by FASD*.” (DR23: page 16)

“*Target all women of reproductive age with the following message of awareness: alcohol, smoking and substance abuse can damage the foetus, so avoid these during pregnancy. Identification of pregnant women at risk; identification of pregnant women aged 35 years or more; identification of pregnant women exposed to teratogens, e.g. alcohol*.” (DR2 page 18)

#### Management targeted clauses

Two of the policy documents contained health-related clauses specifically targeting the management of FASD, these were (1) the Human Genetics Policy Guidelines for the Management and Prevention of Genetic Disorders, Birth Defects and Disabilities [70], and (2) the Mini Drug Master Plan [73].

“*Identification and development of appropriate interventions for individuals and families affected by FASD*.” (DR23: page 16)

“*Offer early detection of FAS, with appropriate referral of affected individuals and their parents for counselling and care. Rehabilitation of disabilities and psychosocial support of affected individuals and their families*.” (DR2: page 18)

#### Blanket clauses

These clauses, although of more general nature, were ascribed to the prevention and management of FASD in the selected policy documents.

#### Prevention blanket clauses

Blanket educational-related clauses that could be attributed to the prevention of FASD were identified in seven policy documents, namely (1) the National Strategy for the Prevention and Management of Alcohol and Drug Use amongst Learners in Schools [74], (2) the City of Cape Town Alcohol Drug Strategy [75], (3) the Western Cape Alcohol-related Harms Reduction: White Paper [71], (4) the National Child and Adolescent Mental Health Policy Guidelines [72], (5) the National Adolescent and Youth Health Policy [76], (6) the National Drug Master Plan [77], and (7) the Anti-substance Abuse Programme of Action [78]. The following illustrative clauses were taken from two of the documents.

“*Primary prevention: Implement school-based alcohol and drug use prevention programmes including life skills training as part of life skills/orientation subject. Implement information and awareness campaigns. Implement co-curricular activities and safety interventions such as peer education clubs. Implement drug-free sports programmes. Involve families and communities*.” (DR13: page VI and 23)

“*Intensified campaigns to educate people about substance abuse. Educational campaigns to inform and educate people, in particular, young people, about the dangers of alcohol and drug abuse*.” (DR15: page 86)

The Guidelines for Maternity Care in South Africa [79], the City of Cape Town Alcohol Drug Strategy [75], the Prevention and Treatment for Substance Abuse Act [80], the Western Cape Alcohol-related Harms Reduction: White Paper [71], and the National Drug Master Plan [77] contained health-related blanket clauses that could be ascribed to the prevention of FASD. The two selected clauses exemplify the nature of these generic prevention intentions.

“*At first visit, take a full and relevant history including medical conditions, including psychiatric problems, and previous operations; use of alcohol, tobacco and other substances; family and social circumstances*.” (DR1: page 35)

*“The identification of risky behaviour that is associated with and predisposes people to substance abuse; the detection of conditions such as poverty and other environmental factors that contribute to crime and the abuse of substances. Identification of individuals, families and communities at risk; screening for problematic substance use to facilitate early detection and appropriate interventions; enabling affected persons to recognise the warning signals of substance abuse. Identification of individuals, families and communities at risk; screening for problematic substance use to facilitate early detection and appropriate interventions*.” (DR7: page 16)

Eight policy documents contained clauses attributed to blanket social considerations ascribed to the prevention of FASD. These documents comprised (1) the National Strategy for the Prevention and Management of Alcohol and Drug Use Amongst Learners in Schools [74], (2) the National Drug Master Plan [77], (3) the Prevention and Treatment for Substance Abuse Act [80], (4) the Western Cape Alcohol-related Harms Reduction: White Paper [71], (5) the National Child and Adolescent Mental Health Policy Guidelines [72], (6) the City of Cape Town Alcohol Drug Strategy [75], (7) the Anti-substance Abuse Programme of Action [78], and (8) the National Liquor Policy [81].

“*Establishment and provision of community-based services: provide professional and lay support within the home environment; establish recreational, cultural and sports activities to divert young people from substance abuse*.” (DR7: page 24)

“*Comprehensive prevention programmes: implementation of universal and targeted programmes, such as those covering life skills. Multiple approaches to prevention across different disciplines, e.g. youth development programmes, sport and skills development*.” (DR15: page 86)

#### Management blanket clauses

Blanket educational clauses that could be attributed to the management of FASD were contained in 10 of the 33 identified policy documents, namely (1) the National Integrated School Health Policy [82], (2) the Policy on Screening, Identification, Assessment and Support [83], (3) the National Integrated Early Childhood Development Policy [84], (4) the National Development Plan 2030 [85], (5) the Education White Paper 5 on Early Childhood Education [86], (6) the Special Needs Education: Building an Inclusive Education and Training System (Education White Paper 6) [87], (7) the National Child and Adolescent Mental Health Policy Guidelines [72], (8) the Guidelines for Responding to Learner Diversity in the Classroom [88], (9) the Guidelines to Ensure Quality Education and Support in Special Schools and Special School Resource Centres [89], and (10) the National Adolescent and Youth Health Policy [76]. Two of these clauses are represented below.

“*Five specific support provision areas are identified: Specialist support staff, assistive devices, specialised equipment and teaching and learning support materials, curriculum differentiation to meet the individual needs of learners*.” (DR16: page 8)

“*Provide a stimulating environment to enhance the development of the child. Provide special classes in normal schools and special schools for intellectual children and adolescents*.” (DR11: page 23)

The Human Genetics Policy Guidelines for the Management and Prevention of Genetic Disorders, Birth Defects and Disabilities [70], the National Integrated Early Childhood Development Policy [84], and the National Development Plan 2030 [85] contained blanket clauses relating to the health management of FASD. The subsequent clauses exemplify this.

“*Screening and early detection of disability, diagnostic and therapeutic support service, 24-hour service and specialist support*.” (DR2 page 22)

“*Nutrition intervention for pregnant women and young children. Ensure household food and nutrition security*.” (DR30 page 30)

Social consideration blanket clauses attributed to the management of FASD were found in eight documents, including (1) the National Development Plan 2030, (2) National Disability Policy [90], (3) the National Child and Adolescent Mental Health Policy Guidelines [72], (4) the National Integrated Early Childhood Development Policy [84], (5) the Guidelines to Ensure Quality Education and Support in Special Schools and Special School Resource Centres [89], (6) the National Youth Policy [91], (7) the Special Needs Education: Building an Inclusive Education and Training System (Education white paper 6) [87], and (8) the Human Genetics Policy Guidelines for the Management and Prevention of Genetic Disorders, Birth Defects and Disabilities [70]. Illustrated are two of the clauses from the documents.

“*Strengthen youth service programmes and introduce new, community-based programmes to offer young people life-skills training, entrepreneurship training and opportunities to participate in community development programmes. Efforts to ensure relevant and accessible skills development programmes for people with disabilities, coupled with equal opportunities for their productive and gainful employment, must be prioritised*.” (DR30: page 30)

“*Ensure that an intellectually disabled child or adolescent is in the hands of an effective carer; where possible, ensure that the natural parents are the primary caregivers for intellectually disabled children and adolescent; provide ongoing emotional and (if necessary) material and human resource support for the parents and other caregivers; provide a stimulating environment to enhance the development of the child*.” (DR11: page 23)

## Discussion

Herein, we aimed to identify the targeted and generic clauses that could be attributed to the prevention and management of FASD in various related policy documents in South Africa. The study also serves as an update to the South African FASD policy document review conducted in 2008 [17]. The significance of this review lies in its capacity to shed light on the extent to which FASD issues are considered in existing South African policy documents, as there is no specific policy document currently addressing FASD. Furthermore, the targeted and generic clauses identified could inform the development of a guideline for policy to address FASD in South Africa.

Our findings confirmed that South Africa still does not have a specific policy addressing FASD since the observation was first made by Randall-Nkosi et al. in 2008 [17]. In their review, only two policy documents used the term FAS, with FASD not being reported at all. However, based on our review, there has been an increase in the use of FAS and FASD, with 12 policy documents mentioning these terms since 2008. The increase in the number of policy documents mentioning FAS and FASD could be seen as an indication that more consideration is given to the issue of FASD across various South African government departments. However, we cannot categorically say that this increase in consideration has translated to an improvement in service delivery. Services are still generic in nature with gaps in policy [18, 21, 42], particularly those involving the coordination of services, communication and collaboration among departments, as previously reported [17, 19]. Therefore, we believe that developing a multi-sectoral policy for FASD could improve communication and collaboration among the relevant departments and, consequently, the coordination of FASD-related services.

Most of the policy documents identified in our review contained targeted and blanket or generic clauses that could be attributed to the prevention and management of FASD. We found a distinct improvement on what was reported in 2008, although none of the policy documents provided a comprehensive, holistic and detailed strategy on how FASD could be prevented and managed. For example, the National Liquor Policy (2016) provided an excellent contextualisation and detailed account of the problem of FASD in South Africa but contained only generic approaches for the prevention of FASD. Although the policy on Screening, Identification, Assessment, and Support (2014) does not mention the terms FAS and FASD, the document provided detailed information on screening, identification, assessment and support for learners identified with learning difficulties; this information could benefit individuals with FASD as it is not unusual for them to present learning difficulties [1]. However, one of the shortcomings of the Policy on Screening, Identification, Assessment, and Support (2014) is that it does not holistically address issues (medical and social) other than the educational issue. Therefore, we suggest a more comprehensive approach to the management of FASD.

Some of the prevention strategies identified in the policy documents were efforts proposed to reduce accessibility and availability of alcohol [21]. These exertions include banning alcohol advertisement, increasing the legal drinking age, regulating alcohol trading hours and liquor outlets, and enforcement of liquor laws. These strategies were similar to those identified in previous studies of policy requirements for FASD in South Africa [18, 21, 42]. These strategies have been found to be effective in reducing accessibility and availability of alcohol. In two studies [43, 44], an association between alcohol availability, rates of alcohol consumption and alcohol-related harms were found. Evidence also indicates that increasing the minimum purchase age of alcohol reduces alcohol-related harms [45]. However, enforcing liquor laws remains a prominent problem in South Africa as the government is struggling to balance the economic benefits of liquor against public health hazards [46]. In the Western Cape, for example, there are approximately 25,000 unlicensed/illegal liquor stores making alcohol available and accessible at a lower price [15]. Therefore, in order to reduce accessibility and availability of alcohol, we recommend that the government should take the enforcement of liquor laws seriously.

Other sections that were identified and could be ascribed to the prevention of FASD were education and creating awareness on the dangers of alcohol to different groups (children, adolescents and adults) and at different levels (schools and communities). Education and awareness formed part of the policy requirements for FASD in previous studies [18, 21, 42]. Education alone does not really modify behaviour as reported in the literature [47, 48]. However, research has also shown that education and awareness have the potential to reduce the prevalence of FASD in areas where they are low [49]. Therefore, we suggest education- and awareness-targeted FASD prevention programmes in areas where education and awareness of FASD are low [49, 50]. In areas where education and awareness on FASD are high, we suggest personalised interventions delivered through innovative approaches such as motivational interviewing and case management [51, 52]. These personalised interventions could be targeted at pregnant women and women of childbearing age.

In our review, we identified proposed clauses to enhance the cessation of drugs and alcohol use, including addressing pregnant women at clinics and in communities. These services include counselling, treatment and rehabilitation services, aftercare and reintegration services, screening, identification, and support and skills development, all of which align with the policy requirements for preventing FASD previously identified [18, 21, 42]. The mentioned services are notable, and if routinely carried out, could assist individuals who are struggling with alcohol, especially pregnant women and those of reproductive age, to minimise or cease drinking during pregnancy. However, research has shown that only 45% of health professionals who care for pregnant women routinely ask about alcohol use and only 25% of them provide information on the consequences of alcohol use in pregnancy [53]. Therefore, we recommend training of health professionals on how best to assist women with an alcohol problem.

Addressing alcohol consumption during pregnancy to prevent FASD is essential. Therefore, it will require asking about alcohol use and providing information on the consequences of its use during pregnancy with specific reference to FASD. The latter should be made a compulsory routine for health professionals caring for pregnant women. Inadequate access to treatment for substance abusers in South Africa has also been reported [54–56], particularly in historically disadvantaged communities [55, 56]. Furthermore, barriers such as limited allocation of resources, stigma and negative beliefs about treatment, hamper substance users from seeking treatment [57, 58]. The inadequate access and barriers to treatment have the potential to influence alcohol use before and during pregnancy. Therefore, we propose an increase in access to treatment. In addition, barriers to treatment specifically for alcohol users should be addressed.

We discovered various levels of support and social security systems for individuals with disabilities outlined in some of the policy documents, which have been reported as part of policy requirements for FASD [21]. The support aims at improving learning participation, meeting the learning needs, assisting parents, and providing a supportive learning environment. Though these support programmes were not specifically targeted at individuals with FASD, those living with the disorder could benefit from them if they qualified. The social security system could also be beneficial to individuals with FASD but would depend on whether they qualify based on the types of primary and secondary characteristics they exhibit. If the South African government can recognise FASD as a disability irrespective of the types of primary and secondary characteristics presented, it would be a first step in the right direction. Therefore, we suggest the inclusion of individuals with FASD and their parents/caregivers in social services allowing them to maximise their potential and improve developmental outcomes [59].

Other strategies we identified in the reviewed policy documents could be ascribed to the management of individuals with FASD, including screening, assessment, identification and support, early childhood development programmes, provision of inclusive education, differentiation and modification of assessment and curriculum, skills development, and training of teachers. These aligned with the policy requirements identified for the management of FASD in South Africa [18, 21, 42]. All these mentioned strategies may be useful if they are directly targeting FASD [59]. Although a screening protocol exists for conditions posing learning barriers, including FASD, there is no national surveillance for FASD in South Africa [17, 18], which means the situation is very similar to what was reported in 2008.

According to the literature, many individuals with FASD only get discovered when they present with other co-morbidities such as intellectual difficulties and conduct disorder, while some of them are misdiagnosed or never diagnosed [60]. Therefore, they are unable to receive the necessary treatment in a timely manner, suggesting the need for national surveillance and specialist diagnostic and support services, as earlier advocated by previous studies [17, 18]. Training all professionals on how to manage FASD should be considered essential, as FASD requires a multidisciplinary team to diagnose and manage [33]. Nevertheless, no specialised training on FASD for health professionals is in place, as reported by the participants in a study published in 2018 [21]. Various studies have indicated the need for training professionals on FASD [53, 61–66]. We suggest that skills development should also be considered paramount as most of the individuals with FASD may not be able to complete formal education [67, 68].

The continuous rise in the prevalence of FASD in South Africa remains a public health concern as reported in the literature [5, 18]. Therefore, it is pertinent for the South African government to respond to this epidemic in a more coordinated and comprehensive manner by designing a specific policy and targeted interventions aimed at preventing and managing FASD. Reducing the prevalence of FASD will directly or indirectly contribute to the reduction in crime rate, risky sexual behaviour, learning problems, alcohol-related harms and other secondary FASD disabilities [67, 68]. We advocate that the South African government should also replicate the success recorded in HIV/AIDS for FASD [22–24]. When developing a policy for FASD, new approaches to policy development on FASD should be considered. We propose that the South African government should learn from the approaches adopted by other governments such as Australia; an example is the Development of Action Australian Plan on FASD (2013–2016), which has led to an increase in government funding, expansion of prevention programmes, and the establishment of specialist FASD diagnostic services [69].

The strength of this study lies in the fact that we accessed several policy documents for FASD-related conditions from multiple departments to show the extent to which FASD has been considered in those policies. The limitation of this study is that we might not have included all the relevant policy documents because we used only two search engines and received only a few responses from the people we contacted. Another limitation is that we only included the latest policy documents of those published in series.

## Conclusion

Herein, we revealed the existence of targeted and generic clauses that could be attributed to FASD. There has also been an increase in the number of policy documents mentioning FASD in the last 10 years. However, the sustained high prevalence of FASD in South Africa reported in the literature calls for holistic and comprehensive approaches to tackle this problem.

## Additional files

Additional file 1: List of documents containing clauses for FASD. (DOCX 22 kb)

Additional file 2: Targeted and blanket clauses for the prevention and management of FASD. (DOCX 29 kb)

## Abbreviations

FAS: fetal alcohol syndrome
FASD: fetal alcohol spectrum disorders

## References

[1] Centers for Disease Control and Prevention (CDC) (2018). Basics about FASD.

[2] Centers for Disease Control and Prevention, National Center on Birth Defects and Developmental Disabilities. Alcohol Use in Pregnancy. 2018*.* www.cdc.gov/ncbddd/fasd/alcohol-use.html. Accessed 16 Jul 2018.

[3] National Organization on Fetal Alcohol Syndrome (2018). National Organization of Fetal Alcohol Syndromehttps.

[4] Lange S, Probst C, Gmel G (2017). Global prevalence of fetal alcohol spectrum disorder among children and youth. JAMA Pediatr.

[5] Olivier L, Curfs LMG, Viljoen DL (2016). Fetal alcohol spectrum disorders: prevalence rates in South Africa. South African Med J.

[6] May P, De Vries M, Marais A-S (2017). Replication of high fetal alcohol spectrum disorders prevalence rates, child characteristics, and maternal risk factors in a second sample of rural communities in South Africa. Int J Environ Res Public Health.

[7] London L (1999). The ‘dop’ system, alcohol abuse and social control amongst farm workers in South Africa: a public health challenge. Soc Sci Med.

[8] Williams G (2016). Slaves, workers, and wine: the ‘Dop system’ in the history of the Cape Wine industry, 1658–1894. J South Afr Stud.

[9] World Health Organizayion (2014). Global Status Report on Alcohol and Health.

[10] South African Government. South African Demographic and Health Survey (SADHS). 2016. https://www.gov.za/documents/south-african-demographic-and-health-survey-sadhs. Accessed 16 Apr 2019.

[11] Popova S, Lange S, Probst C (2017). Prevalence of alcohol consumption during pregnancy and fetal alcohol spectrum disorders among the general and Aboriginal populations in Canada and the United States. Eur J Med Genet.

[12] Cloete L, Ramugondo E (2015). ‘I drink’: mothers’ alcohol consumption as both individualised and imposed occupation. South African J Occup Ther.

[13] Petersen Williams P, Jordaan E, Mathews C (2014). Alcohol and other drug use during pegnancy among women attending midwife obstetric units in the Cape Metropole, South Africa. Adv Prev Med.

[14] Vythilingum B, Roos A, Faure SC, Geerts L, Stein DJ (2012). Risk factors for substance use in pregnant women in South Africa. South African Med J.

[15] Charman A, Petersen L (2016). Comment on the Western Cape alcohol-related harms reduction policy green paper charman and petersen, sustainable livelihoods foundation.

[16] Esper LH, Furtado EF (2014). Identifying maternal risk factors associated with Fetal Alcohol Spectrum Disorders: a systematic review. Eur Child Adolesc Psychiatry.

[17] Rendall-Mkosi K, London L, Adnams C, et al. Fetal Alcohol Spectrum Disorder in South Africa: Situational and Gap Analysis. Pretoria: UNICEF. 2008. www.unicef.org/southafrica/SAF_resources_fetalalcohol.pdf. Accessed 14 Sep 2017.

[18] Jacobs L, Steyn N, Labadarios D (2013). Mind the Gap’: Observations in the Absence of Guidelines for Alcohol Abstinence among Expectant Women in South Africa.

[19] Adnams CM (2017). Fetal alcohol spectrum disorder in Africa. Curr Opin Psychiatry.

[20] Adebiyi BO, Mukumbang FC, Okop KJ (2018). A modified Delphi study towards developing a guideline to inform policy on fetal alcohol spectrum disorders in South Africa: a study protocol. BMJ Open.

[21] Adebiyi BO, Mukumbang FC, Cloete LG (2018). Exploring service providers’ perspectives on the prevention and management of fetal alcohol spectrum disorders in South Africa: a qualitative study. BMC Public Health.

[22] Wouters E, van Rensburg H, Meulemans H (2010). The National Strategic Plan of South Africa: what are the prospects of success after the repeated failure of previous AIDS policy?. Health Policy Plan.

[23] Hopkins KL, Doherty T, Gray GE (2018). Will the current National Strategic Plan enable South Africa to end AIDS, tuberculosis and sexually transmitted infections by 2022?. South Afr J HIV Med.

[24] Johnson LF, Dorrington RE, Moolla H (2017). Progress towards the 2020 targets for HIV diagnosis and antiretroviral treatment in South Africa. South Afr J HIV Med.

[25] Chibango C (2013). South Africa’s HIV and AIDS policy and legislation: an analysis. Greener J Med Sci.

[26] World Health Organization (2011). Fetal Alcohol Syndrome: Dashed Hopes, Damaged Lives. Bull World Health Organ.

[27] Benz J, Rasmussen C, Andrew G (2009). Diagnosing fetal alcohol spectrum disorder: history, challenges, and future directions. Paediatr Child Health.

[28] Fitzpatrick JP, Latimer J, Olson HC (2017). Prevalence and profile of neurodevelopment and fetal alcohol spectrum disorder (FASD) amongst Australian Aboriginal children living in remote communities. Res Dev Disabil.

[29] Calhoun F, Attilia ML, Spagnolo PA (2006). National Institute on Alcohol Abuse and Alcoholism and the study of fetal alcohol spectrum disorders. The International Consortium. Ann Ist Super Sanita.

[30] Foundation for Alcohol Education and Research. The Australian Fetal Alcohol Spectrum Disorders Action Plan 2013-2016. 2013. https://www.fare.org.au/wp-content/uploads/research/FARE-FASD-Plan.pdf. Accessed 5 Feb 2019.

[31] Watkins RE, Elliott EJ, Halliday J (2013). A modified Delphi study of screening for fetal alcohol spectrum disorders in Australia. BMC Pediatr.

[32] Public Health Agency of Canada. Framework: Fetal Alcohol Spectrum Disorder (FASD): A Framework for Action - Canada.ca. www.canada.ca/en/public-health/services/reports-publications/fetal-alcohol-spectrum-disorder-fasd-framework-action/fetal-alcohol-spectrum-disorder-fasd-a-framework-action-1.html#d. Accessed 8 Jun 2018.

[33] Cook JL, Green CR, Lilley CM (2016). Fetal alcohol spectrum disorder: a guideline for diagnosis across the lifespan. CMAJ.

[34] Bower Carol, Elliott Elizabeth J, Zimmet Marcel, Doorey Juanita, Wilkins Amanda, Russell Vicki, Shelton Doug, Fitzpatrick James, Watkins Rochelle (2017). Australian guide to the diagnosis of foetal alcohol spectrum disorder: A summary. Journal of Paediatrics and Child Health.

[35] Bertrand J, Floyd LL, Weber MK, Fetal Alcohol Syndrome Prevention Team, Division of Birth Defects and Developmental Disabilities, National Center on Birth Defects and Developmental Disabilities, Centers for Disease Control and Prevention (CDC). Guidelines for identifying and referring persons with fetal alcohol syndrome. MMWR Recomm Rep. 2005;54(RR-11):1–14. Available from: http://www.ncbi.nlm.nih.gov/pubmed/16251866. 16251866

[36] Substance Abuse and Mental Health Services Administration (SAMHSA). SAMHSA’s New Treatment Improvement Protocol focuses on FASD. SAMHSA - Substance Abuse and Mental Health Services Administration. 2018. https://www.samhsa.gov/fetal-alcohol-spectrum-disorders-fasd-center. Accessed 16 Jul 2018.

[37] Bowen Glenn A. (2009). Document Analysis as a Qualitative Research Method. Qualitative Research Journal.

[38] Corbin J, Strauss A (2008). Basics of Qualitative Research (3rd ed.): Techniques and Procedures for Developing Grounded Theory.

[39] Erlingsson C, Brysiewicz P (2017). A hands-on guide to doing content analysis. African J Emerg Med.

[40] Gale NK, Heath G, Cameron E (2013). Using the framework method for the analysis of qualitative data in multi-disciplinary health research. BMC Med Res Methodol.

[41] Miles M, Huberman A (1994). Qualitative Data Analysis: An Expanded Sourcebook.

[42] Adebiyi BO, Mukumbang FC, Cloete LG (2019). Policymakers’ perspectives towards developing a guideline to inform policy on fetal alcohol spectrum disorder: a qualitative study. Int J Environ Res Public Health.

[43] Bowers Y, Rendall-Mkosi K, Davids A (2014). Liquor outlet density, deprivation and implications for foetal alcohol syndrome prevention in the Bergriver municipality in the Western Cape, South Africa. South African Geogr J.

[44] Donnelly N, Poynton S, Weatherburn D (2006). Liquor outlet concentrations and alcohol-related neighbourhood problems.

[45] Wagenaar AC, Toomey TL (2002). Effects of minimum drinking age laws: review and analyses of the literature from 1960 to 2000. J Stud Alcohol Suppl.

[46] Parry CDH (2010). Alcohol policy in South Africa: a review of policy development processes between 1994 and 2009. Addiction.

[47] Larimer ME, Cronce JM (2007). Identification, prevention, and treatment revisited: individual-focused college drinking prevention strategies 1999–2006. Addict Behav.

[48] Logan DE, Marlatt GA (2010). Harm reduction therapy: a practice-friendly review of research. J Clin Psychol.

[49] Chersich MF, Urban M, Olivier L (2012). Universal prevention is associated with lower prevalence of fetal alcohol spectrum disorders in Northern Cape, South Africa: a multicentre before-after study. Alcohol Alcohol.

[50] Parry CDH, Gossage JP, Marais A-S (2012). Comparison of baseline drinking practices, knowledge, and attitudes of adults residing in communities taking part in the FAS prevention study in South Africa. Afr J Drug Alcohol Stud.

[51] de Vries M, Joubert B, Cloete M (2015). Indicated prevention of fetal alcohol spectrum disorders in South Africa: effectiveness of case management. Int J Environ Res Public Health.

[52] Hanson JD, Miller AL, Winberg A (2013). Prevention of alcohol-exposed pregnancies among nonpregnant American Indian women. Am J Health Promot.

[53] Payne J, Elliott E, D’Antoine H (2005). Health professionals’ knowledge, practice and opinions about fetal alcohol syndrome and alcohol consumption in pregnancy. Aust N Z J Public Health.

[54] Lutchman S (2015). Insufficient access to substance abuse treatment centres for illicit drug users and its potential effect on a foetus: a breach of the right to access health care services. Law Democr Dev.

[55] Myers B, Parry CD (2005). Access to substance abuse treatment services for black South Africans: findings from audits of specialist treatment facilities in Cape Town and Gauteng: original article. Afr J Psychiatry.

[56] Myers BJ, Louw J, Pasche SC (2010). Inequitable access to substance abuse treatment services in Cape Town, South Africa. Subst Abuse Treat Prev Policy.

[57] Myers B, Louw J, Fakier N (2008). Alcohol and drug abuse: removing structural barriers to treatment for historically disadvantaged communities in Cape Town. Int J Soc Welf.

[58] Myers B, Fakier N, Louw J (2009). Stigma, treatment beliefs, and substance abuse treatment use in historically disadvantaged communities. Afr J Psychiatry.

[59] Reid N, Dawe S, Shelton D (2015). Systematic review of fetal alcohol spectrum disorder interventions across the life span. Alcohol Clin Exp Res.

[60] Popova S, Lange S, Shield K (2016). Comorbidity of fetal alcohol spectrum disorder: a systematic review and meta-analysis. Lancet.

[61] Mukherjee R, Wray E, Curfs L (2015). Knowledge and opinions of professional groups concerning FASD in the UK. Adopt Foster.

[62] Flannigan K, Pei J, Stewart M (2018). Fetal alcohol spectrum disorder and the criminal justice system: a systematic literature review. Int J Law Psychiatry.

[63] Cox LV, Clairmont D, Cox S (2008). Knowledge and attitudes of criminal justice professionals in relation to fetal alcohol spectrum disorder. Can J Clin Pharmacol.

[64] Van Schalkwyk I, Marais S (2017). Educators’ relational experiences with learners identified with fetal alcohol spectrum disorder. South African J Educ.

[65] Scheepers P (2009). Educators’ knowledge of and attitudes toward fetal alcohol spectrum disorder.

[66] Passmore HM, Mutch RC, Burns S (2018). Fetal alcohol spectrum disorder (FASD): Knowledge, attitudes, experiences and practices of the Western Australian youth custodial workforce. Int J Law Psychiatry.

[67] Clark E, Lutke J, Minnes P (2004). Secondary disabilities among adults with fetal alcohol spectrum disorder in British Columbia. J FAS Int.

[68] Streissguth AP, Barr HM, Kogan J, Bookstein FL. Understanding the Occurrence of Secondary Disabilities in Clients with Fetal Alcohol Syndrome and Fetal Alcohol Effects. 1996. p. 1–72. http://lib.adai.uw.edu/pubs/bk2698.pdf.

[69] Reid N (2018). Fetal alcohol spectrum disorder in Australia: what is the current state of affairs?. Drug Alcohol Rev.

[70] Human Genetics Policy Guidelines for the Management and Prevention Of Genetic Disorders, Birth Defects and Disabilities. 2001. https://www.westerncape.gov.za/text/2003/humangenetics.pdf. Accessed 15 Apr 2019.

[71] The Western Cape Alcohol-related Harms Reduction: White Paper. 2017. https://www.westerncape.gov.za/text/2017/September/white_paper_alcohol-related_harms_reduction.pdf. Accessed 15 Apr 2019.

[72] The National Child and Adolescent Mental Health Policy Guidelines. 2003. https://www.health-e.org.za/wp-content/uploads/2013/11/child_mental_health.pdf. Accessed 15 Apr 2019.

[73] Mini Drug Master Plan. http://policyresearch.limpopo.gov.za/handle/123456789/889. Accessed 24 Aug 2018.

[74] National Department of Basic Education. National Strategy for the Prevention and Management of Alcohol and Drug Use Amongst Learners in Schools. 2013. http://www.kzneducation.gov.za/Portals/0/snes/NationalStrategy_UNICEF_PRINT_READY.pdf. Accessed 15 Apr 2019.

[75] City of Cape Town. The City of Cape Town Alcohol Drug Strategy. 2014. https://libguides.lib.uct.ac.za/c.php?g=182363&p=1581389. Accessed 16 Apr 2019.

[76] National Department of Health. National Adolescent & Youth Health Policy. 2017. https://www.saferspaces.org.za/resources/entry/national-adolescent-youth-health-policy-2017. Accessed 16 Apr 2019.

[77] South Africa Government. National Drug Master Plan 2013-2017. 2013. https://www.gov.za/documents/national-drug-master-plan-2013-2017. Accessed 16 Apr 2019.

[78] Anti-Substance Abuse Programme of Action. 2011. https://www.thedti.gov.za/business_regulation/docs/nla/Anti_Substance_Abuse.pdf. Accessed 24 Aug 2018.

[79] National Department of Health. The Guidelines for Maternity Care in South Africa. 2015. https://www.google.com/search?client=firefox-b-d&q=The+Guidelines+for+Maternity+Care+in+South+Africa. Accessed 16 Apr 2019.

[80] South African Government. Prevention of and Treatment for Substance Abuse Act. 2009. https://www.gov.za/documents/prevention-and-treatment-substance-abuse-act. Accessed 16 Apr 2019.

[81] South Africa Government. Liquor Act: National Liquor Policy. 2016 https://www.gov.za/documents/liquor-act-national-liquor-policy-30-sep-2016-0000. Accessed 16 Apr 2019.

[82] National Department of Basic Education. The National Integrated School Health Policy. 2012. https://serve.mg.co.za/content/documents/2017/06/14/integratedschoolhealthpolicydbeanddoh.pdf. Accessed 16 Apr 2019.

[83] National Department of Basic Education. The Policy on Screening, Identification, Assessment and Support. 2014. https://wcedonline.westerncape.gov.za/Specialised-ed/documents/SIAS-2014.pdf. Accessed 16 Apr 2019.

[84] The National Integrated Early Childhood Development Policy. 2015. http://www.gauteng.gov.za/government/departments/education/Policies/2015/National intergrated Early Childhood development Policy2015.pdf. Accessed 16 Apr 2019.

[85] National Development Plan 2030. 2013. https://www.gov.za/documents/national-development-plan-2030-our-future-make-it-work. Accessed 16 Apr 2019.

[86] South Africa Government. Education White Paper 5 on Early Childhood Education. 2001. https://www.gov.za/documents/education-white-paper-5-early-childhood-education. Accessed 16 Apr 2019.

[87] South Africa Government. Special Needs Education: Education White Paper 6. 2001. https://www.gov.za/documents/special-needs-education-education-white-paper-6. Accessed 16 Apr 2019.

[88] National Department of Basic Education. The Guidelines for Responding to Learner Diversity in the Classroom. 2011. https://www.education.gov.za/Portals/0/Documents/Publications/GUIDELINESFORRESPONDINGTOLEARNERDIVERSITYTHROUGHCAPS(FINAL).pdf?ver=2016-02-24-110910-340. Accessed 16 Apr 2019.

[89] National Department of Basic Education. The Guidelines to Ensure Quality Education and Support in Special Schools and Special School Resource Centres. 2008. https://wcedonline.westerncape.gov.za/Specialised-ed/documents/Guidelines-to-ensure-QualityEducationandSupport.pdf. Accessed 16 Apr 2019.

[90] National Disability Policy. https://www.westerncape.gov.za/assets/departments/social-development/national_disability_policy.pdf. Accessed 16 Apr 2019.

[91] South African Government. National Youth Policy. 2015. https://www.gov.za/documents/national-youth-policy-2015-2020-8-jun-2015-0000. Accessed 16 Apr 2019.

