# Supplementary material for: To what extent is Fetal Alcohol Spectrum Disorder considered in policy-related documents in South Africa? A document review
Source: Health Res Policy Syst. 2019 Apr 29;17:46. doi: 10.1186/s12961-019-0447-9 (PMC6489263; doi:10.1186/s12961-019-0447-9)
Supplement: Supplementary file 1 — List of documents containing clauses for FASD. (DOCX 22 kb) [file 12961_2019_447_MOESM1_ESM.docx]

**Additional file 1: List of documents containing clauses for FASD**

|  | **Title** | **Author/year** | **Description** |
| --- | --- | --- | --- |
| DR1 | Guidelines for Maternity Care in South Africa (GFMC) | National Department of Health, South Africa (2015). | This document describes maternity care in South Africa. It consists of seventeen chapters. Chapter one provides an overview of the current situation and data on maternal care in South Africa. Chapter four contains subsections on preconception care (ask about the use of tobacco, alcohol and other recreational drugs) and first antenatal visit (take a full and relevant history including, use of alcohol, tobacco, and other substances) |
| DR2 | Human Genetics Policy Guidelines for the Management and Prevention of Genetic Disorders, Birth Defects, and Disabilities (HGPG) | National Department of Health, South Africa (2001). | This document describes the prevention and management of genetic disorders, birth defects and disabilities in South Africa. Chapter one detailed situational analysis of genetic disorders and birth defects in South Africa. It comprises of fourteen chapters. Chapter four consists of strategies for prevention of the genetic disorder. Fetal alcohol syndrome was list one of the priority disorders. |
| DR3 | Special Needs Education: Building an Inclusive Education and Training System (Education white paper 6) (SNEB) | National Department of Education, South Africa (2001). | This document describes an inclusive education and training system in South Africa. It contains four chapters. Chapter one provides context to inclusive education in South Africa. The document detailed how every learner can be assisted in an inclusive manner with exclusion, either in the mainstream schools or special schools |
| DR4 | National Liquor Policy (NALP) | Department of Trade and Industry, South Africa (2016) | This document outlines the policy recommendations intended to amend the Liquor Act, 59 of 2003 (“the Act”). This document consists of five sections. Section three (purpose and problem statement) outlines the socio-economic impact of liquor and other costs of alcohol abuse. |
| DR5 | City of Cape Town Alcohol Drug Strategy 2014 -2017 CCTA) | City of Cape Town (2014 -2017) | This document outlines the strategy to reduce the health, economic and social burden caused by alcohol and other drugs (AOD) abuse in Cape Town. It consists of six sections. |
| DR6 | Gauteng Liquor Bill (GALB) | Gauteng Government (2011) | The purpose of this document is to: (1) facilitate responsible attitudes towards the production, distribution, promotion, marketing, advertising, sale and consumption of liquor; (2) ensure that appropriate measures are in place to reduce the harm caused by the consumption of alcohol including reducing the per capita consumption of alcohol; and (3) facilitate the entry and empowerment of new entrants in the liquor industry in Gauteng province. It consists of nine sections |
| DR7 | Prevention and Treatment for Substance Abuse Act (PTSA) | South Africa Government (2008) | This document consists of eleven chapters. It provide: (1) a comprehensive national response for the combating of substance abuse; (2) mechanisms aimed at demand and harm reduction in relation to substance abuse through prevention, early intervention, treatment and reintegration programmes; (3) for the registration and establishment of treatments centres and halfway houses; (4) for the committal of persons to and from treatment centres and for their treatment, rehabilitation and skills development in such a treatment centres; (5) for the establishment of the central drug authority; and (6) for matters connected therewith |
| DR8 | South African Children Act (SACA) | South Africa Government (2005) | This document consists of twenty-two chapters. It was developed: (1) to give effect to certain rights of children as contained in the Constitution; (2) to set out principles relating to the care and protection of children; to define parental responsibilities and rights; (3) to make further provision regarding children’s courts; (4) to provide for the issuing of contribution orders; (5) to make new provision for the adoption of children; (6) to provide for inter-country adoption; (7) to give effect to the Hague Convention on Inter-country Adoption; (8) to prohibit child abduction and to give effect to the Hague Convention on International Child Abduction; (9) to provide for surrogate motherhood; to create certain new offences relating to children; and (10) to provide for matters connected therewith. |
| DR9 | Anti-substance Abuse Programme of Action (AAPA) | South Africa Government (2011-2016) | The programme of Action was developed based on resolutions taken during the 2nd Biennial Anti Substance Summit held from the 15th to the 17th March 2011 at the International Convention Centre in KwaZulu Natal Province. |
| DR10 | National Liquor Act (NALA) | South Africa Government (2003) | This Act was developed to establish national norms and standards in order to maintain economic unity within the liquor industry; to provide for essential national standards and minimum standards required for the rendering of services; to provide for measures to promote co-operative government in the area of liquor regulation, and to provide for matters connected therewith. It consists of eight chapters. Chapter one comprises of definitions, interpretation, objects, and application of the Act. |
| DR11 | National Child and Adolescent Mental Health Policy Guidelines (NCAM) | National Department of Health, South Africa (2003) | This document serves as a framework for establishing mental health services for children and adolescents at national, provincial and local levels of health care within the Primary Health Care approach using an inter-sectoral approach. They have been developed in relation to other South African policies, legislation, and treaties that address themselves to the enhancement of survival as well the protection and development of children and adolescents. The document adopts a holistic approach in addressing the various risk and protective factors that can affect the mental health of children and adolescents. These causal factors can exist in the physical, emotional and social domains of the human being. |
| DR12 | National Adolescent and Youth Health Policy (NAYH) | National Department of Health, South Africa (2017) | This document was formulated: (1) to promote the mental health and wellbeing of adolescents and youth in schools, families, and communities; (2) to reduce substance abuse and violence; (3) to provide comprehensive, integrated sexual and reproductive health services; (4) to test and treat for HIV/AIDS and TB and keep people within the health system; (5) to support nutrition and healthier diet; (6) to strengthen health systems to respond to the needs of adolescents and youth. |
| DR13 | National Strategy for the Prevention and Management of Alcohol and Drug Use Amongst Learners in Schools (NSPM) | National Department of Basic Education (2103) | This document was developed in order to provide an integrated and comprehensive strategy that can guide the Department of Basic Education (DBE) and the provinces in a coordinated effort to address alcohol and drug use in schools. It comprises of five chapters. |
| DR14 | Education White Paper 5 on Early Childhood Education (EWPE) | National Department of Basic Education (2001) | This document consists of six sections. It focuses on expanding early childhood development (ECD) provision, correcting the imbalances in the provision, ensuring equitable access and improving the quality and delivery of ECD programmes. |
| DR15 | National Drug Master Plan (NDMP) | National Department of Social Development (2013 - 2017) | This document complements the work of the Inter-Ministerial Committee on Alcohol and Drug Abuse by guiding and monitoring the actions of the government departments to reduce the demand for and supply of drugs and the harm associated with their use and abuse. It comprises of ten chapters |
| DR16 | Policy on Screening, Identification, Assessment, and Support (PSIA) | National Department of Basic Education (2014) | This document was created to provide a policy framework for the standardization of the procedures to identify, assess and provide programmes for all learners who require additional support to enhance their participation and inclusion in school. It contains seven chapters |
| DR17 | Western Cape Liquor Act (WCLA) | Western Cape Government (2008) | This act was developed to provide for the licensing for the retail sale and the micro-manufacture of liquor in the Western Cape Province, and to provide for incidental matters. It consists of thirteen chapters. |
| DR18 | Western Cape Alcohol-related Harms Reduction: White Paper (WCAH) | Western Cape Government (2016) | This document was developed to provide interventions to contribute to the reduction of alcohol-related harms in the Western Cape. A further purpose is to provide for ancillary matters to increase the efficiency and effectiveness of supplementary supporting structures that are related to alcohol-related harms reduction. |
| DR19 | South African Schools Act (SASA) | National Department of Basic Education (1996) | This document was created: (1) to provide for a uniform system for the organization, governance, and funding of schools; (2) to amend and repeal certain laws relating to schools; and (3) to provide for matters connected therewith. It comprises of seven chapters. |
| DR20 | South Africa Mental Health Act (SAMH) | South Africa Government (2002) | This document was developed; (1) to provide for the care, treatment and rehabilitation of persons who are mentally ill; (2) to set out different procedures to be followed in the admission of such persons; (3) to establish Review Boards in respect of every health establishment; (4) to determine their powers and functions; (5) to provide for the care and administration of the property of mentally ill persons; (6) to repeal certain laws; and (7) to provide for matters connected therewith. |
| DR21 | South African Social Security Agency Act, (SASS) | South Africa Government (2004) | This document was formulated: (1) to provide for the establishment of the South African Social Security Agency as an agent for the administration and payment of social assistance; (2) to provide for the prospective administration and payment of social security by the Agency and the provision of services related thereto; and (3) to provide for matters connected therewith. It consists of six chapters. |
| DR22 | National Integrated School Health Policy (NISH) | National Department [Basic Education and Health] (2012) | This document describes how to the improvement of the general health of school-going children as well as the environmental conditions in schools and address health barriers to learning in order to improve education outcomes of access to school, retention within school and achievement at school. |
| DR23 | Mini Drug Master Plan (MDMP) | National Department of Health (2011/2012 – 2013/2014) | This document was developed to reduce the health, economic and social burden caused by substance abuse in South Africa, through the provision of targeted demand and harm reduction interventions provided by the National Department of Health, thereby supporting the implementation of the National Drug Master Plan. |
| DR24 | National mental health policy framework and strategic plan (NMHP) | National Department of Health (2013-2020) | This document was formulated to enable the mental health and well-being of all South Africans from infancy to old age, through the provision of evidence-based, affordable and effective promotion, prevention, and treatment and rehabilitation interventions. In partnerships between providers, users, carers and communities, the human rights of people with mental illness will be upheld; they will be provided with care and support, and they will be integrated into normal community life. |
| DR25 | National Integrated Early Childhood Development Policy (NIEC) | National Department of Social Development (2015) | This document comprises of twelve chapters. The purpose of this Policy is: (1) to provide an overarching multi-sectoral enabling framework of early childhood development services, inclusive of national, provincial and local spheres of government; (2) to define a national comprehensive early childhood development programme and support, with identified essential components; (3) to identify the relevant role players and their roles and responsibilities for the provision of the various components of early childhood development services; and (4) to establish national integrated early childhood development leadership and coordinating structure. |
| DR26 | National Youth Policy (NAYP) | South Africa Government (2015 – 2020) | This document describes the intention of government toward youth in South Africa. it is comprised of background, introduction, rationale, context, and legislation, defining and profiling South Africa’s youth, situation analyses and challenges, policy proposals, and conclusion. |
| DR27 | White Paper on the Rights of Persons with Disabilities (WPRP) | National Department of Social Development (2015) | This document is intended to accelerate transformation and redress with regard to full inclusion, integration, and equality for persons with disabilities. It consists of seven parts |
| DR28 | National Plan of Action for Children (NPAC) | National Department of Women, children and with Disabilities (2012-2017) | This document was developed to promote the realization of children’s rights to survival, development, protection, participation and to mobilize resources on all levels. |
| DR29 | Western Cape School Act (WCSA) | Western Cape Government (1997) | This document was formulated to provide for a uniform education system for the organization, governance, and funding of all schools; consequentially to repeal certain laws relating to schools, and to make provision for the specific educational needs of the Western Cape province |
| DR30 | National Development Plan 2030 (NDPL) | South Africa National Government (2013) | This document consists of fifteen chapters. Each chapter in this plan contains a range of targets and proposals. Some of these are general statements of policy intent. Others are policy proposals actions and processes that need to occur. The plan is to change the life chances of millions of people in South Africa. |
| DR31 | National Disability Policy (NADP) | National Department of Social Development | This document outlines the: context, background, aim, purpose, scope, policy and legislative mandates, key principles, roles, responsibilities, approach, structures for Implementation, resource allocations and monitoring and evaluation. It aims to facilitate the provision of integrated social services to people with disabilities |
| DR32 | Guidelines to Ensure Quality Education and Support in Special Schools and Special School Resource Centres (GEQE) | National Department of Basic Education (2014) | This document was developed to ensure quality education and support in special schools outlines some of the requirements for a functional special school. The information in this guideline is organized under the following headings: legislative and policy framework; curriculum management and implementation; personnel supply and qualifications; and Infrastructure and hostel accommodation |
| DR33 | Guidelines for Responding to Learner Diversity in the Classroom (GRLD) | National Department of Basic Education (2011) | This document is intended to provide teachers, principals, subject advisors, administrators, school governors, and other personnel, parameters and strategies on how to respond to learner diversity in the classrooms through the curriculum. It comprises of the following sections: understanding diversity in the classroom; responding to diversity through the curriculum; differentiating the curriculum; analyzing, recording and reporting on assessment; and accessing support for curriculum differentiation |
