# Supplementary material for: To what extent is Fetal Alcohol Spectrum Disorder considered in policy-related documents in South Africa? A document review
Source: Health Res Policy Syst. 2019 Apr 29;17:46. doi: 10.1186/s12961-019-0447-9 (PMC6489263; doi:10.1186/s12961-019-0447-9)
Supplement: Supplementary file 2 — Targeted and blanket clauses for the prevention and management of FASD. (DOCX 29 kb) [file 12961_2019_447_MOESM2_ESM.docx]

**Additional file 2:** **Targeted clauses for the prevention and management of FASD**

| Themes | Sub-themes | Codes from various sources |
| --- | --- | --- |
| Prevention | Education | The continuation of education programmes on FASD. The Western Cape Government (WCG) will continue to focus on education programmes on FASD in collaboration with strategic partners specialising in the field, with the aim of expanding the programme. Current initiatives include screening participants and providing psychosocial therapy and life-skills training. (DR18: page 62)  Educate all women regarding the deleterious effects of alcohol on the foetus. Educate all women to avoid alcohol throughout pregnancy. (DR2: page 18)  Education of women of child-bearing age who are not on birth control of the risks alcohol consumption poses to foetal development (through life-skills education in schools and broader education campaigns). (DR18: page 51) |
|  | Health | Public efforts to improve health, nutrition, education and self-reliance, particularly of women; avoidance of unintended pregnancies, and proper birth spacing through access to contraception and other methods of family planning; improved access to, and quality of, pre-natal care and genetic counselling; avoidance of exposure to teratogens (e.g. alcohol) during pregnancy. (DR2: page 15)  Improve the detection rate for alcohol and other drug abuse at antenatal clinics, and provide the appropriate services to reduce the incidence of foetal alcohol syndrome. (DR11: page 21)  Provide interventions at antenatal clinics to reduce the likelihood of alcohol-exposed pregnancies. Included in the measures are: (ii) encouraging earlier uptake of antenatal services for pregnant women and expecting fathers, and ongoing screening of alcohol use for women attending antenatal clinics, and (iii) active case management of mothers who attend antenatal clinics who are at higher risk of an alcohol-exposed pregnancy. The latter has been found to help women at risk to either stop drinking or cut down on drinking during pregnancy, leading to a reduced risk of FASD. (DR18: page 51) |
|  | Social | Identification and development of appropriate interventions for individuals and families affected by FASD. (DR23: page 16)  Target all women of reproductive age with the following message of awareness: alcohol, smoking and substance abuse can damage the foetus, so avoid these during pregnancy. Identification of pregnant women at risk; identification of pregnant women aged 35 years or more; identification of pregnant women exposed to teratogens, e.g. alcohol. (DR2: page 18) |
| Management | Education |  |
|  | Health | Identification and development of appropriate interventions for individuals and families affected by FASD. (DR23: page 16)    Offer early detection of FAS, with appropriate referral of affected individuals and their parents for counselling and care. Rehabilitation of disabilities and pyscho-social support of affected individuals and their families. (DR2: page 18) |
|  | Social |  |

**Table 2: Blanket clauses that can be attributed to the prevention of FASD**

| Themes | Sub-themes | Codes from various sources |
| --- | --- | --- |
| Prevention | Education | Early Detection: Partake in early warning systems. Train educators & parents to identify warning signs. Train educators & district officials to conduct drug testing. Implement drug testing in schools where there is reasonable suspicion that learners are using drugs. Set up systems to ensure continuation of education during treatment (DR13: page VI and 24)  Primary Prevention: Implement school-based alcohol and drug use prevention programmes including life skills training as part of life skills/orientation subject. Implement information & awareness campaigns. Implement co-curricular activities and safety interventions such as peer education clubs. Implement drug free sport programmes. Involve families & communities. (DR13: 24)  Implement school-based alcohol and drug use prevention programmes including life skills training as part of life skills / orientation subject. (DR13: page 23)  Early intervention: training of social workers; brief intervention. (DR5: page 8)  Train all enforcement officials in all aspects of liquor legislation, liquor control and liquor enforcement. Develop a guide and make it available to prosecutors and other state officials. (DR18: page 40)  Train primary health care practitioners and educators in basic counselling skills and trauma support. Ensure that children and adolescents who suffer from post-traumatic stress disorder receive, in addition to other treatment approaches, counselling from a professional who has the appropriate training and experience. Improve the substance-related component in the training of people who are already counselling children and adolescents. Improve training of health personnel (especially nursing staff) in the detection, diagnosis, and management of patients suffering from substance abuse, both in their specialised professional training and their continuing professional development. (DR11: pages 21 and 22)  The curriculum must include accessible and practical information about HIV/AIDS and TB, mental health, sexual and reproductive health, nutrition and healthy weight, substance abuse and violence prevention. (DR12: page 8)  Prioritise the Provincial substance abuse forum (PSAF) and Local drug action committees (LDACs) as platforms for integration, referral pathways and reciprocal communication. Continue and strengthen the Western Cape Education Department (WCED) education and awareness interventions. Develop norms and standards for school-based prevention programmes. Leverage the after-school space for education and awareness targeted at youth and the provision of, or referral to interventions. Support education and awareness at the post-school education level. Promote a clean fun campaign. Mainstream education and awareness in all WCG departments. Promote and strengthen education and awareness programmes with stakeholders Improve the reach and ease of access to education and awareness material. Expand and strategically direct addiction care education courses. (DR18: pages 58-62)  Intensified campaigns to educate people about substance abuse. Educational campaigns to inform and educate people, in particular young people, about the dangers of alcohol and drug abuse. (DR15: page 86, DR9 page 12)  Implement information & awareness campaigns. (DR13: page VI)  Educate parents, teacher and other adults close to children and adolescents of the importance of not explicitly or implicitly condoning cigarette smoking, inappropriate alcohol use and illicit drugs. (DR11: page 20) |
|  | Health | The following information should be provided to all women of childbearing age before conception: the risks to the foetus when the mother takes teratogenic medications during pregnancy. Certain essential information must be provided to all pregnant women, verbally and (where possible) in the form of written or illustrated cards or pamphlets. This includes: abuse of alcohol, tobacco and recreational drugs. (DR1: pages 34 and 38)  At first visit, take a full and relevant history including: medical conditions, including psychiatric problems, and previous operations; use of alcohol, tobacco and other substances; family and social circumstances. (DR1: page 35)  Women at risk for having a child with a birth defect or genetic disorder; refer as early as possible in the pregnancy for counselling regarding management and the performance of prenatal tests. Check list of risk factors requiring referral or hospital delivery: known substance abuse including alcohol.  (DR1: pages 34 and 43)  Screening: high risk individual, pregnant women, street people, mental health and testing people in high risk work. (DR5: page 8)  The identification of risky behaviour that is associated with and predisposes people to substance abuse; the detection of conditions such as poverty and other environmental factors that contribute to crime and the abuse of substances. Identification of individuals, families and communities at risk; screening for problematic substance use to facilitate early detection and appropriate interventions; enabling affected persons to recognise the warning signals of substance abuse. Identification of individuals, families and communities at risk; screening for problematic substance use to facilitate early detection and appropriate interventions. (DR7: page 16)  Provide programmes for screening, provision of information, brief motivational interventions and appropriate treatment services for hazardous and harmful drinking in primary health care settings, including antenatal clinics, and in emergency units. Provide interventions at antenatal clinics to reduce the likelihood of alcohol-exposed pregnancies. Strengthen the to-be-developed early screening and referral services at schools and other institutions of learning, targeting high-risk areas in the province. Specialised services for treatment and brief interventions for youth to be expanded and included at child- and youth-care centres and appropriate health facilities. (DR18: page 50-51)  Developing and implementing multi-disciplinary and multi-modal protocols and practices for integrated diagnosis, treatment and funding of co-occurring disorders for adults, youths and children, e.g. providing for prevention, early detection, treatment and aftercare services, and integrating requisite changes through policy, legislation, protocols and practices. (DR15: page 98) |
|  | Social | Treatment, Care and Support: Establish strong referral systems to access treatment, care & support Create awareness of treatment and counselling services. Address barriers that limit access to treatment amongst learners (e.g. transport, cost and stigma). (DR13: page VI)  Implement co-curricular activities and safety interventions such as peer education clubs. Implement drug free sport programmes. (DR13: page VI)  Ensure equal access to resources, especially for civil society and organisations in rural areas, e.g. recreational facilities, sport facilities, diversion programmes, intellectual development programmes, skills development.(DR15: page 89)  Establishment and provision of community-based services: provide professional and lay support within the home environment; establish recreational, cultural and sports activities to divert young people from substance abuse. (DR7: page 24)  The Department of Social Development (DSD), in collaboration with the Department of Health (DOH) and other stakeholders, will strengthen evidence-based prevention, early intervention, detoxification, treatment and aftercare interventions. (DR18: page 49)  Justice and Constitutional Development; engage in effective co-operation and practical action in addressing the world drug problem on the basis of common and shared responsibility for: increasing international co-operation and following an integrated, multi-disciplinary, mutually reinforcing and balanced approach in demand and supply reduction strategies; strengthening mechanisms for cooperation and co-ordination; and developing methods to facilitate the exchange of experiences and good practice. (DR15: page 105)  Coordination: facilitate collaborations; facilitate referral; involve ward committees. Sector Coordination: facilitate collaborations and support Non-governmental organisation (NGOs) and Community-based organisation (CBO). Sphere Coordination: facilitate inter-government collaborations. (DR5: page 8)  Support the work done by faith based organisations, NGO’s and CBO’s in educating children and adolescents about substance abuse. (DR11: page 20)  Ensure that the social skills that are particularly relevant for substance use are included in existing life skills programmes. (DR11: page 20)  Measures aimed at skills development for individuals, families and communities to enable them to enjoy a better quality of life. (DR7: page 16)  Comprehensive prevention programmes: implementation of universal and targeted programmes, such as those covering life skills. Multiple approaches to prevention across different disciplines, e.g. youth development programmes, sport and skills development. (DR15: page 86)  After-school programmes, youth development and skills and capacity building. (DR5: page 8)  Establish an effective referral system to services provided by the DOH, DSD, other departments and Non-profit organisations (NPOs) to provide long-term treatment, prevention and diversion activities Outpatient care for clients on treatment and rehabilitation services, as well as active outreach into communities, to be strengthened to improve treatment outcomes. (DR18: page 52)  Utilise the Child-to-Child approach and strategies to encourage peer support for behavioural change and support seeking by children and adolescents. Create local group-based support for parental, societal and economic empowerment opportunities for women with children. Support self-help groups and families of children and adolescents with substance use problems. (DR11: page 22)  Provide for support groups for service users and those affected by substance abuse. (DR7: page 24)  Strengthening aftercare services, e.g. providing for prevention, early detection, treatment and aftercare services; integrating requisite changes through policy, legislation, protocols and practices, with emphasis on children, young people and learners. Increasing the provision of rehabilitation and aftercare, e.g. through providing for prevention, early detection, treatment and aftercare services, and integration of requisite changes through policy, legislation, protocols and practices, with special provision for access by all communities. (DR15: page 98)  Aftercare and reintegration that provide for — the integration of people who have undergone the formal treatment episode into their families and communities. (DR7: page 18)  Medical interventions that address the physiological and psychiatric needs of the service user; psycho-social programmes that address the relationships, emotions, feelings, attitudes, beliefs, thoughts and behaviour patterns of service users. (DR7: page 16)  Improve availability of substance-related counselling services at key sites (e.g schools, health facilities, prison, streets. (DR11: page 20)  The community-based model for substance abuse treatment and rehabilitation is expanded. (DR18: page 53)  Increasing the provision of rehabilitation and after care and ensuring that all communities have access to these services. (DR9: page 13)  Progressively increase the coverage of alcohol-related harms interventions at all public-health and social-service facilities in the Western Cape as well as for community action engagement interventions. (DR18: page 28)  Lobby for a national ban on alcohol advertising that is visible to any person under the age of 18 and for restrictions on sports advertising and promotion that links alcohol to aspirational achievement. (DR18: page 25)  The national minimum legal age at which alcohol can be purchased and consumed should be raised from eighteen (18) to twenty one (21) years. (DR4: page 6)  Reducing accessibility of alcohol through raising the legal age for purchasing and public consumption of alcohol from the age of 18 to the age of 21, e.g. through changing policy, legislation, protocols and practice in a harmonised manner nationally; developmental programmes relating to changes; assessing effects of changes. (DR15: page 90)  Banning all sponsorship of sport, recreation, arts, cultural and related events by the alcohol industry, e.g. through changing policy, legislation, protocols and practice in a harmonised manner nationally; running developmental programmes relating to changes; assessing effects of changes. (DR15: page 95)  Prohibit advertising, marketing and promotion of alcohol products and companies at all events organised by the WCG. (DR18: page 27)  Banning all sponsorship by the alcohol industry for sports, recreation, arts and cultural and related events. (DR9: page 13)  All broadcast television channels should advertise at night, from 22:00- 06:00; remove content appealing to youth in alcohol advertising such as using of sport stars, models, etc. Branding of liquor premises and delivery trucks and/ or cars should be prohibited; and counter advertisement which identifies the harmful effects of liquor abuse, for example, car crashes and victims, ailments caused by liquor, family violence and other social ills. (DR4: page 24)  Support the application of (national) levies on marketing and promotional spending to cover alcohol-related harms counter-messaging. (DR18: page 28)  Imposing a mandatory contribution by the liquor industry to a fund that will be dedicated to work to prevent and treat alcohol abuse. (DR9: page 11)  Provincially determine a set maximum limits for trading hours in line with the alcohol-related harms reduction approach of reducing consumption. Provision for exceptions would allow for flexibility based on set criteria, and the relevant authorities would be able to reduce trading hours within the framework. (DR18: page 29)  Imposing restrictions on the time and days of the week that alcohol can be legally sold. These restrictions must be uniform, that is, they must be applicable in all provinces. (DR9: page 11)  The set uniform trading hours within the norms and standards should be integrated in national, provincial and municipal legislation. (DR4: page 25)  Imposing restrictions on the times and days of the week that alcohol can be sold legally, e.g. through changing policy, legislation, protocols and practice in a harmonised manner nationally; developmental programmes relating to changes; assessing effects of changes. (DR15: page 90)  Reduce the availability of alcohol by regulating density of outlets (zoning requirements and population density) and regulating trading days and hours. (DR18: page 29)  Implementing laws and regulations that will reduce the number of liquor outlets, including shebeens, taverns and liquor stores in specific geographical areas. These laws and regulations should include stricter licensing laws and qualifying criteria and specific zoning laws and regulations that will prescribe the locations of different types of economic activity that can take place in residential areas. The zoning laws should for example, ensure that no liquor outlets are located near schools, libraries and places of worship. (DR9: page 11)  Implementing laws and regulations that will reduce the number of liquor outlets including shebeens, e.g. through changing policy, legislation, protocols and practice in a harmonised manner nationally; developmental programmes relating to changes; assessing effects of changes. (DR15: page 91)  Regulating the density of alcohol outlets is an effort to minimise excessive alcohol consumption and related harms. Outlet density may be regulated through licensing and zoning regulations. (DR4: page 25)  Lobby the national government to incentivise the reduction of the ethanol content in alcohol beverages. (DR18: page 33)  Prescribing measures for alcohol containers such as the form of container, warning labels and the percentage alcohol content. (DR9: page 11)  Lobby national government to increase the price of alcohol through increasing excise tax and/or introducing minimum unit pricing and consider a provincial tax. The increased tax revenue would be ring-fenced for alcohol-harms reduction investments. (DR18: page 31)  Prescribing measures for alcohol containers, e.g. through changing policy, legislation, protocols and practice in a harmonised manner nationally; running developmental programmes relating to changes; assessing effects of changes; form of containers; warning labels; percentage of alcohol content. (DR15: page 93)  Lobby the national government to implement a tracking system of liquor products. (DR18: page 33)  Raising of duties and taxes on alcohol products to deter the purchasing of alcohol. The tariffs should be implemented on a sliding scale commensurate with the alcoholic content. (DR9: page 11)  Raising duties and taxes on alcohol products to deter the purchasing of alcohol, e.g. through changing policy, legislation, protocols and practice in a harmonised manner nationally; running developmental programmes relating to changes; assessing effects of changes; implementing sliding-scale tariffs commensurate with alcoholic content. (DR15: page 92)  Take steps to bring some responsible unlicensed liquor outlets into the regulated space in a sustainable and responsible manner to facilitate compliance with minimum requirements. Create awareness of alternative economic opportunities to currently unlicensed outlet owners who cannot be accommodated within the applicable zoning scheme. (DR18: pages 34 and 35)  Identify mechanisms and criteria, working with municipalities that will enable the rezoning of outlets for liquor sales in appropriate residential areas. Prioritise upstream interventions targeting suppliers to the unlicensed liquor industry and the illicit liquor trade. (DR18: page 35)  Change legislation to enable some of the unlicensed outlets to be licensed and therefore regulated. Increase enforcement of under-age drinking laws. Implement innovative strategies to reduce harms from problematic outlets. Promote the involvement of communities themselves through interactive opportunities to access the Western Cape Liquor Authority (WCLA) complaints mechanisms. (DR18: pages 37-39)  Clamp down on unlicensed outlets and the supply of liquor to unlicensed outlets. All spheres of government and relevant departments must contribute to the clamp-down. Information from community-based organisations and structures as well as the enforcement opportunities from municipal zoning schemes, the Western Cape Land Use Planning Act, 2014 (Act 3 of 2014) (LUPA), and the Spatial Planning and Land Use Management Act, 2013 (Act 16 of 2013) (SPLUMA), must be leveraged to aid the law-enforcement agencies. (DR18: page 38)  Lobby for well-prepared police dockets for the prosecution of liquor-related matters by providing evidence of the link between crime and alcohol – and provide evidence, where appropriate, to support the prosecutions process. (DR18: page39) |

**Table 2: Blanket clauses that can be attributed to the management FASD**

| Themes | Sub-themes | Codes from various sources |
| --- | --- | --- |
| management | Education | Learner Assessment and Screening: Assessments during the foundation phase focus primarily on identifying health barriers to learning, as well as identifying children who have or are at risk for long-term health, psychosocial or other problems. The following assessments will be done on all foundation phase learners: conduct vision, speech and basic hearing screening; measurement of height, weight and Body Mass Index (BMI); appropriate nutritional interventions must be planned accordingly; check for fine and gross locomotor problems; conduct oral health screening; screen for chronic illness or long-term health conditions - this includes both communicable diseases (such as TB and HIV/AIDS) as well as non-communicable diseases; and perform a basic mental health and/or psychosocial risk assessment. (DR22: page 13)  Learners in the senior and FET phases should also be screened for weight and body mass index, vision, oral health, chronic illness or long-term health conditions and mental/ psychosocial health issues. (DR22: page 13)  The Screening, Identification, Assessment and Support policy specifically aims to identify (1) the barriers to learning experienced, (2) the support needs that arise from barriers experienced and (3) to develop the support programme that needs to be in place to address the impact of the barrier on the learning process. (DR16: page 4)  Provision of early childhood development services. Provision of comprehensive quality early childhood development programme. Provision of universal developmentally appropriate early learning opportunities for young children from birth. Develop and fund multiple early childhood development service delivery programmes and prioritise home and community based services. Inclusion and support for children with disabilities within all early childhood development programmes. Prioritise vulnerable children to ensure equitable access. (DR25: pages 54-55, 64, 66, 68-69)  Universal access to two years of early childhood development. Increase the quality of education so that all children have at least two years of preschool education and all children in grade 3 can read and write. (DR30: pages 33 and 37)  The organisation of early identification and early intervention services will be a key focus in Early Childhood Development (ECD) in both the 0 to 4-year programmes, as well as in Grade R (DR16: page 19)  Opportunities to develop fine motor skills; encouragement of language through talking, being read to, singing; activities that will develop a sense of mastery; experimentation with pre-writing and pre-reading skills; hands on exploration for learning through action; opportunities for taking responsibility and making choices; encouragement to develop self-control, cooperation and persistence in completing projects; support for their sense of self-worth; opportunities for self-expression; and encouragement of creativity. (DR14: page 38)  Integrated Pre-Grade R programmes for special groups of 4 year olds: the delivery of inclusive and integrated Pre-Reception Year programmes can ensure that all children have significantly improved opportunities for growth and development. This will provide opportunities to foster children’s emotional, social, intellectual, physical, spiritual and moral development and to use play as the primary vehicle for achieving this. (DR14: page 39)  Transforming all aspects of the education system, developing an integrated system of education, infusing ‘special needs and support services’ throughout the system. (DR3: page 6)  Improve the school system, including increasing the number of students achieving above 50 percent in literacy and mathematics, increasing learner retention rates to 90 percent and bolstering teacher training. (DR30: page 33)  Provide a stimulating environment to enhance the development of the child. Provide special classes in normal schools and special schools for intellectual children and adolescents. (DR11: page 23)  Curriculum and practitioner development for pre-Reception Year. Particular care will be taken to ensure that when addressing children’s intellectual developmental needs, practitioners make use of developmentally appropriate practice. (DR14: page 39)  Alternate Assessments Based on Grade-level Attainment of Knowledge (content, concepts and skills) for learners with disabilities or learning difficulties that need testing formats or procedures that provide them with equal opportunities to demonstrate their attainment of content which is at the same grade-level as the general assessment. Target learners can include learners who are blind, have communication difficulties, physical disabilities, learners who are dyslexic or with hearing loss and who need additional time, alternate formats, readers, amanuensis, electronic equipment, etc. as outlined in the policy document, National policy on the conduct, administration and management of the National Senior. (DR33: page 19)  As a teacher gets to know her learners, and as learner differences emerge, assessment needs to become more differentiated. The goal is to meet learners where they are and to help them progress to the next step in their learning. Thus it is a cyclical process: assessment and instruction support and inform each other. (DR33: page 13)  Teachers will incrementally be trained on the various curriculum differentiation methodologies so as to be able to apply the various adaptive and supportive assessment measures in school-based as well as formal assessment. (DR33: page 19)  Training of all ECD practitioners will include a component on the SIAS Policy. (DR16: page 19)  Training in teaching methods; regular and systematic monitoring; adequate support to practitioners; provision of more reading books and improvement of the professional status of ECD practitioners. (DR14: page 26)  Five specific support provision areas are identified: Specialist support staff, assistive devices, specialised equipment and teaching and learning support materials, curriculum differentiation to meet the individual needs of learners. (DR16: page 8)  Specialised support resources, personnel, programmes and facilities that are needed on a lower-frequency basis, are holistic and teacher-focused, more portable and requiring less operational and organisational planning, will be provided at circuit or district level to be accessed by learners at ordinary schools, e.g. learning support, remedial education, assistive devices, counselling, rehabilitation and therapeutic services. (DR16: page 12)  Special Schools must ensure that they support the families of learners enrolled in their schools. Such support may include educational guidance and support, psychological and emotional guidance and support, etc. Schools must advise parents on the best possible career opportunities available to learners. (DR32: page 6)  Schools-based and out of school programme to programme; parenting-support to reduce adolescent problem behaviour, including substance abuse and aggression. (DR12: page 8)  Educational support systems should make use of and promote the establishment of a network of support through the Care and Support for Teaching and Learning (CSTL) framework, which coordinates all existing services, including other government departments, community services, private professionals, non-government organisations (NGOs), disabled people organisations (DPOs), early intervention providers and community-based rehabilitation services. (DR16: page 18)  The Screening, Identification, Assessment and Support Policy aims to facilitate the shift from individual learner disability as the driving organiser for support provision to that of the range, nature and level of support programmes, services, personnel and resources that will be made available for special and ordinary schools to increase learner participation in the learning process. (DR16: page 12) |
|  | Health | Screening and early detection of disability, diagnostic and therapeutic support service, 24 hour service and specialist support. (DR2: page 22)  Referral system: referral to social and Welfare Support Services. (DR2: page 24)  Health care and nutrition programmes; social protection programmes. (DR25: pages 56-57)  Nutrition intervention for pregnant women and young children. Ensure household food and nutrition security. (DR30: pages 33 and 37) |
|  | Social | Strengthen youth service programmes and introduce new, community-based programmes to offer young people life-skills training, entrepreneurship training and opportunities to participate in community development programmes. Efforts to ensure relevant and accessible skills development programmes for people with disabilities, coupled with equal opportunities for their productive and gainful employment, must be prioritised. (DR30: pages 33 and 52)  Developing and implementing empowerment programmes for People with disabilities on issues pertaining to Life and social skills, positive self-image and self-perception, positive inter-personal relations and communication, coping and parenting skills and understanding and comprehending relevant policies and available social services. (DR31: page 32)  Ensure that an intellectual disabled child or adolescent is in the hands of an effective carer; where possible, ensure that the natural parents are the primary caregivers for intellectually disabled children and adolescent; provide ongoing emotional and (if necessary) material and human resource support for the parents and other caregivers; provide a stimulating environment to enhance the development of the child. (DR11: page 23)  Support for pregnant women, new mothers/fathers and children under 2 years of age. Parent support programmes; opportunities for learning. (DR25: pages 58, 59, 62)  Work collaboratively with community organisations and structures including disabled people’s organisations, parent organisations, teacher unions, NGOs, Disabled people’s organisation (DPOs), traditional healers, parents, grandparents and caregivers in providing teaching and learning support. Work with the community on advocacy and awareness-raising programmes aimed at changing negative attitudes towards people with disabilities and supporting the implementation of an inclusive education system. (DR32: page 21)  The Department of Sport and Recreation, together with the Department of Cooperative Governance and Traditional Affairs, should provide a plan on how sport and recreation facilities will be established maintained and protected from vandals. (DR26: page 24)  Fostering holistic and integrated support provision through intersectoral collaboration. (DR3: page 6)  To establish inter-sectoral collaborations with relevant stakeholders such as the Departments of Education, Labour, Welfare (Social Development), the private sector, NPOs and CBOs. Collaboration with other Departments (e.g. Education) and sub-directorates (e.g. mental health) Community and NGO liaison. (DR2: page 11)  Entrench a social security system covering all working people, with social protection for the poor and other groups in need, such as children and people with disabilities. (DR30: pages 34)  Developing service delivery norms and standards to ensure the protection and promotion of the rights of People with Disabilities. Developing a technical guide on procedures to promote better understanding to the service providers and beneficiaries, of the social security system. Developing and implementing a communication strategy that will inform people with disabilities of social security services available to them, thereby increasing their access to the services. (DR31: page 31) |
